# Cardiac cement embolism and asymptomatic pulmonary embolism caused by percutaneous vertebroplasty for osteoporotic vertebral fracture: a case report

**DOI:** 10.3389/fsurg.2024.1464049

**Published:** 2024-11-25

**Authors:** Yang Yang, Qi Fei, Gong Qian Long, Wu Bo, Feng Ye Jun, Zhang Rong, Huang Kui

**Affiliations:** Department of Orthopaedics, The First Affiliated Hospital of Yangtze University, Jingzhou, China

**Keywords:** OVFC, PKP, PVP, intracardiac cement emboli, pulmonary emboli

## Abstract

**Background:**

As society ages, the incidence of osteoporotic vertebral compression fractures steadily rises. Procedures like percutaneous kyphoplasty (PKP) and percutaneous vertebroplasty (PVP) have proven effective in significantly relieving pain in patients with these fractures. While PKP and PVP are minimally invasive, complications can still occur. However, most complications are not clinically significant, with cement leakage being the most common.

**Case presentation:**

We present the case of a patient with an osteoporotic vertebral compression fracture who underwent percutaneous kyphoplasty (PKP) and percutaneous vertebroplasty (PVP). On the night following the procedure, the patient experienced transient discomfort in the chest, which resolved on its own. A chest CT scan the next day revealed a 5 cm arc-shaped high-density shadow near the right atrium, along with multiple high-density lung spots. After consulting with cardiothoracic surgery, interventional vascular surgery, and radiology experts, and discussing options with the patient and their family, a thoracotomy was recommended to remove the bone cement from the heart. However, the attempt was unsuccessful. Despite this, the patient made a good recovery and was successfully discharged.

**Conclusions:**

Vascular leakage of bone cement is a potentially life-threatening complication of PKP/PVP, and it warrants careful attention.

## 1 Introduction

As the population ages, the incidence of osteoporotic vertebral compression fractures continues to rise (1). Leading to increased cases of low back pain and restricted mobility, which can significantly impact a patient's quality of life. Percutaneous kyphoplasty (PKP) and percutaneous vertebroplasty (PVP) have proven effective in relieving pain associated with these fractures. These minimally invasive procedures allow patients to regain mobility early, reducing the risk of complications such as pneumonia, deep vein thrombosis, and pressure ulcers.

Despite their benefits, PKP and PVP are not without risks. Cement leakage remains the most common complication, with leakage occurring into veins, paravertebral soft tissues, the intervertebral disc, or the spinal canal, potentially affecting the foraminal or epidural spaces (2). According to a systematic review, the leakage rates for bone cement are 39.3% for PVP and 28.6% for PKP (3). While most instances of leakage are not clinically significant, asymptomatic bone cement pulmonary embolism is relatively common in patients undergoing these procedures. However, intracardiac cement embolism is a rare but potentially life-threatening complication (4). Even small emboli can be fatal if they enter the right atrium or ventricle, causing cardiac perforation or pericardial tamponade, rather than being directed into the pulmonary circulation (5).

In this report, we present a case of a patient with an osteoporotic vertebral compression fracture who underwent PKP/PVP surgery. The patient experienced transient chest discomfort the night following the procedure. A chest CT scan conducted the next day revealed intracardiac cement emboli and bone cement pulmonary emboli. Although a thoracotomy was attempted to remove the embolized cement, the extraction was unsuccessful. To better understand and reduce the risk of such complications, we conducted a thorough review of the existing literature.

## 2 Case report

**Figure 1:**
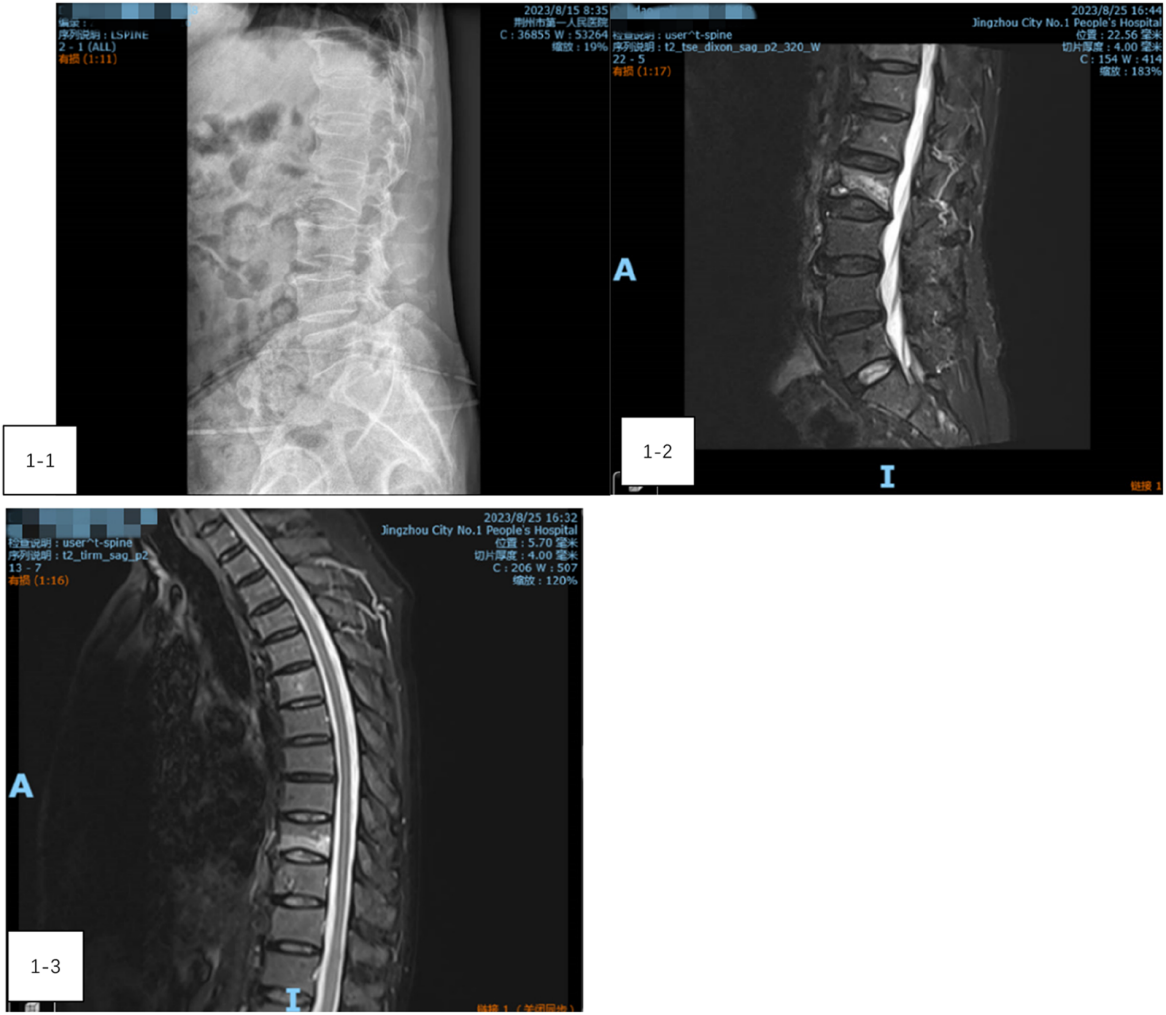
(1-1) A lumbar x-ray confirmed a compression fracture of the L2 vertebra. (1-2) A lumbar MRI further revealed a compression fracture at L2. (1-3) Thoracic vertebra MRI showing bone edema at the T9 vertebra.

**Figure 2:**
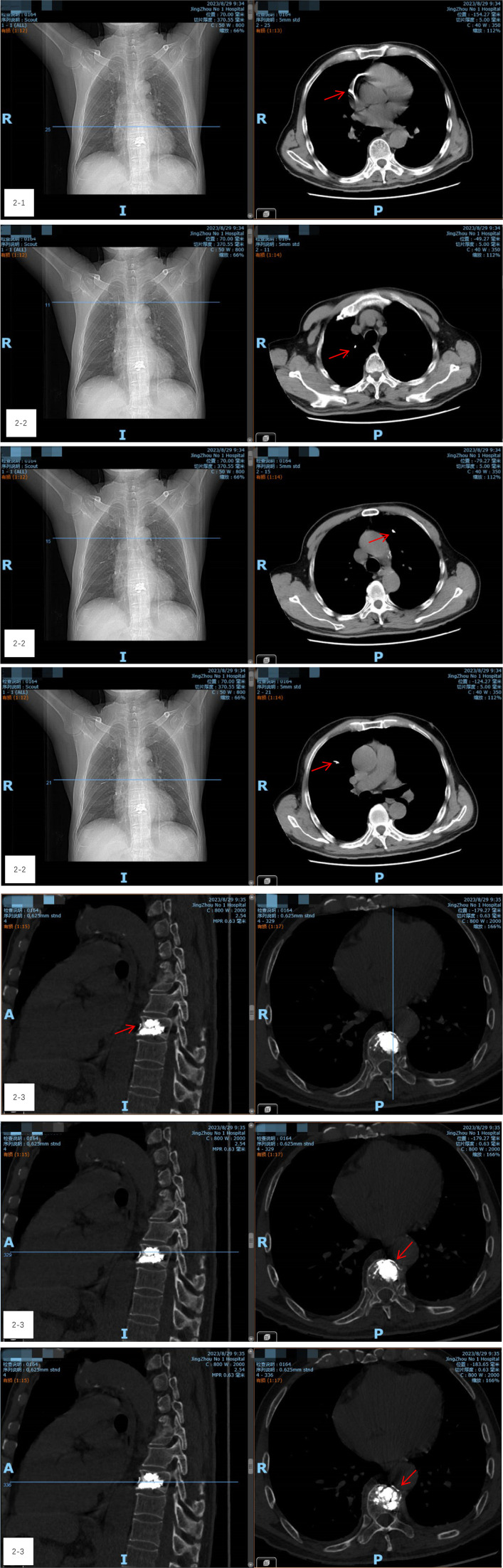
(2-1) A chest CT scan revealed a 5 cm arc-shaped high-density shadow near the right atrium. (2-2) CT shows multiple high-density spots in the lungs. (2-3) A cord-like high-density shadow was detected in the left anterior region of the T9 vertebra.

**Figure 3:**
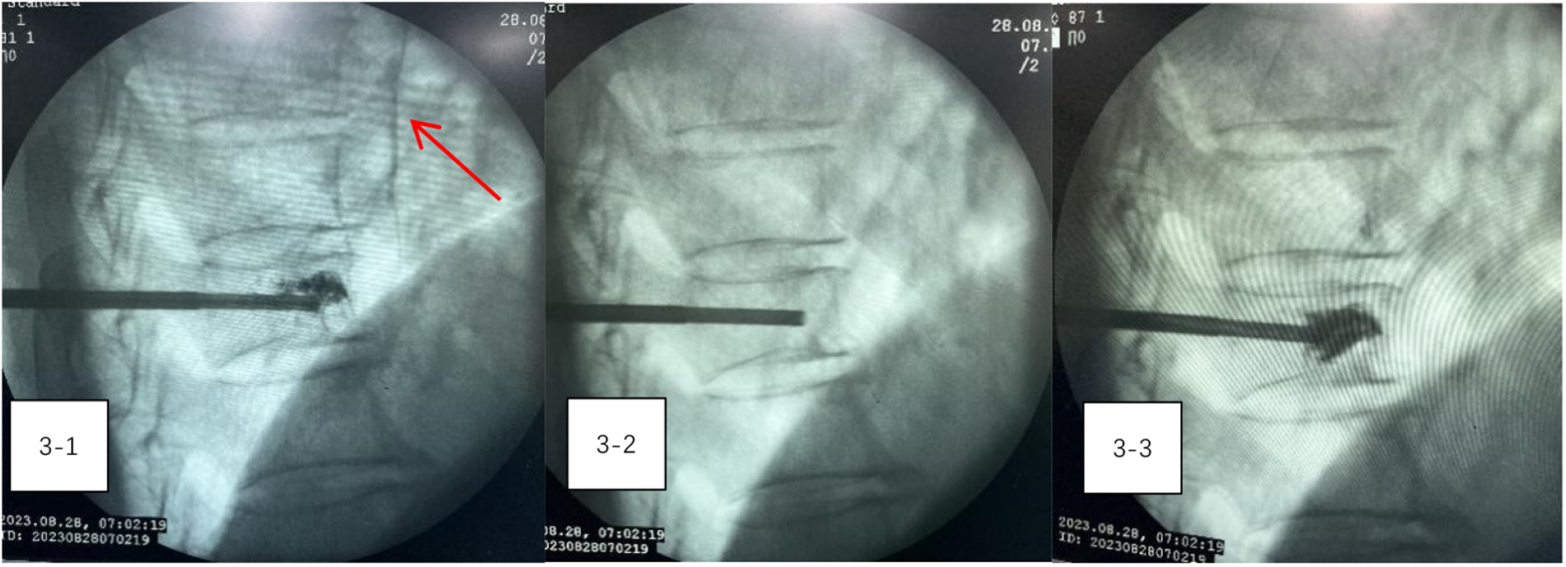
(3-1–3-3) A comparison of pre-and post-injection x-rays showed no elongated high-density shadow before the injection, which appeared after re-injecting 0.5 ml of cement.

A 73-year-old male presented with low back pain and restricted movement for 10 days. His medical history included hypertension and type 2 diabetes, both well-controlled. Before admission, a lumbar x-ray confirmed a compression fracture of the L2 vertebra (Figure 1-1). After admission, a thoracic and lumbar MRI further revealed a compression fracture at L2 and bone edema in the T9 vertebra (Figures 1-2, 1-3). Preoperative evaluations showed no contraindications for surgery. Given the presence of only bone edema at T9 without significant compression, the patient underwent percutaneous kyphoplasty (PKP) for the L2 vertebra and percutaneous vertebroplasty (PVP) for T9 under general anesthesia.

During the procedure, the patient remained stable, with no notable changes in blood pressure, heart rate, or oxygen levels. However, on the night following surgery, he experienced transient precardiac discomfort that resolved on its own. A chest CT scan performed the next day revealed a 5 cm arc-shaped high-density shadow near the right atrium (Figure 2-1) and multiple high-density spots in the lungs (Figure 2-2). Additionally, a cord-like high-density shadow was detected in the left anterior region of the T9 vertebra (Figure 2-3). These findings suggested that bone cement leakage had led to foreign bodies in the heart and bone cement pulmonary embolism. Upon reviewing intraoperative x-rays, it was noted that during the T9 PVP, a long, high-density shadow appeared anterior to the vertebral body on the lateral radiograph after injecting 0.5 ml of bone cement. A comparison of pre-and post-injection x-rays showed no elongated high-density shadow before the injection, which appeared after re-injecting 0.5 ml of cement (Figures 3-1–3-3). This indicated that the bone cement likely leaked into the paravertebral venous system during surgery, ultimately migrating to the heart and causing both intracardiac foreign bodies and pulmonary embolism.

To clarify the nature of the high-density shadow in the heart and determine the best course of action, consultations were held with cardiothoracic surgery, interventional vascular surgery, and radiology departments. Imaging specialists conducted a detailed analysis using axial, sagittal, and coronal views of the mediastinal and bone windows, concluding that the high-density shadows in the heart and lungs were indeed due to bone cement and not imaging artifacts. However, the presence of a long foreign body in the right atrium, near the inferior vena cava, raised concerns about the risk of heart perforation. Due to the smooth surface of the cement, removal via interventional techniques was not feasible.

**Figure 4:**
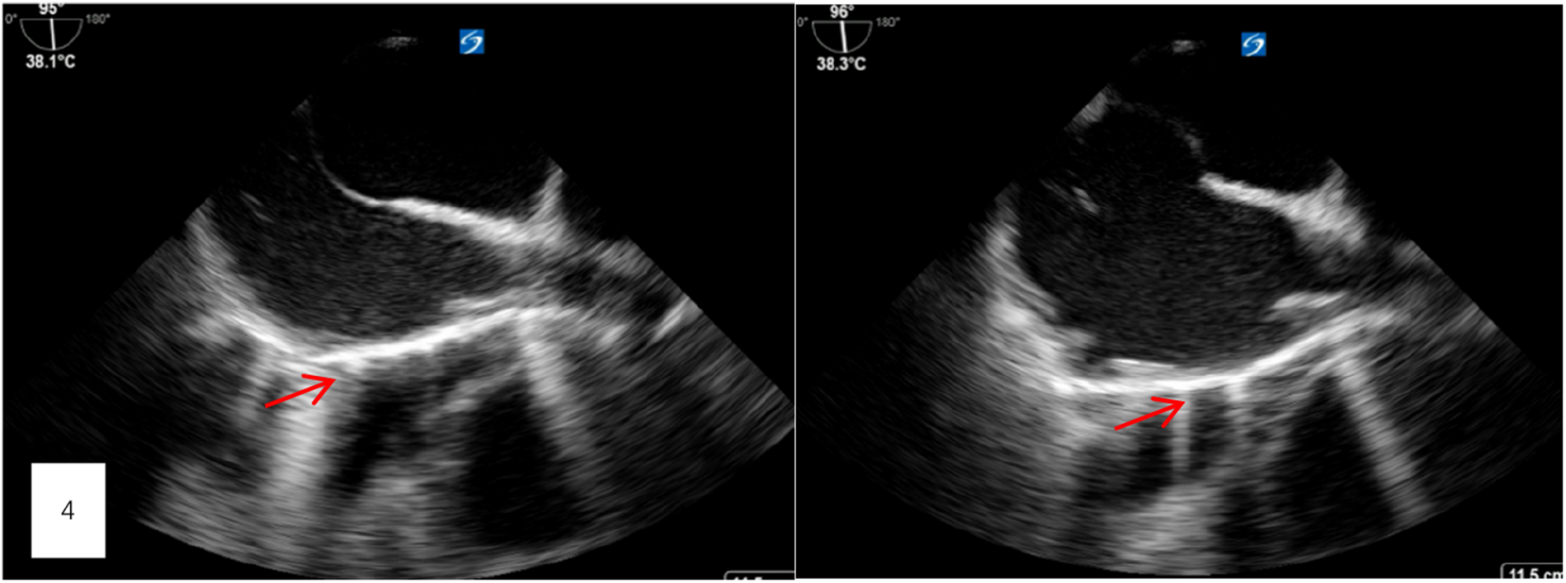
Preoperative transesophageal ultrasound, conducted after general anesthesia, confirmed the presence of a foreign body in the right atrium, measuring approximately 5 cm in length.

After thorough discussions with the patient and his family, a decision was made to proceed with a thoracotomy to remove the bone cement from the heart. On the evening of the first postoperative day, a thoracotomy was performed. Preoperative transesophageal ultrasound, conducted after general anesthesia, confirmed the presence of a foreign body in the right atrium, measuring approximately 5 cm in length (Figure 4). A small amount of pericardial fluid was noted. Still, no obvious foreign body was visible on the heart's surface. Careful exploration of the superior and inferior vena cava revealed no foreign bodies in the right atrium. However, transesophageal ultrasound continued to indicate a foreign body near the right atrium, leading to a decision to perform a cardiotomy with exploration under cardiopulmonary bypass.

Once cardiopulmonary bypass was established, the right atrium was incised, and the upper and lower vena cava were carefully examined. No foreign body was detected. The intraoperative transesophageal ultrasound then identified the foreign body in the right ventricle, close to the outflow tract. The surgical team induced cardiac arrest incised the atrial septum, and placed left ventricular drainage. Upon exploring the right ventricle and the pulmonary artery root through the tricuspid valve, no significant foreign body was identified. Further exploration of the left atrium and left ventricle also revealed no foreign bodies. A final check with transesophageal ultrasound found no additional evidence of an intracardiac foreign body.

**Figure 5:**
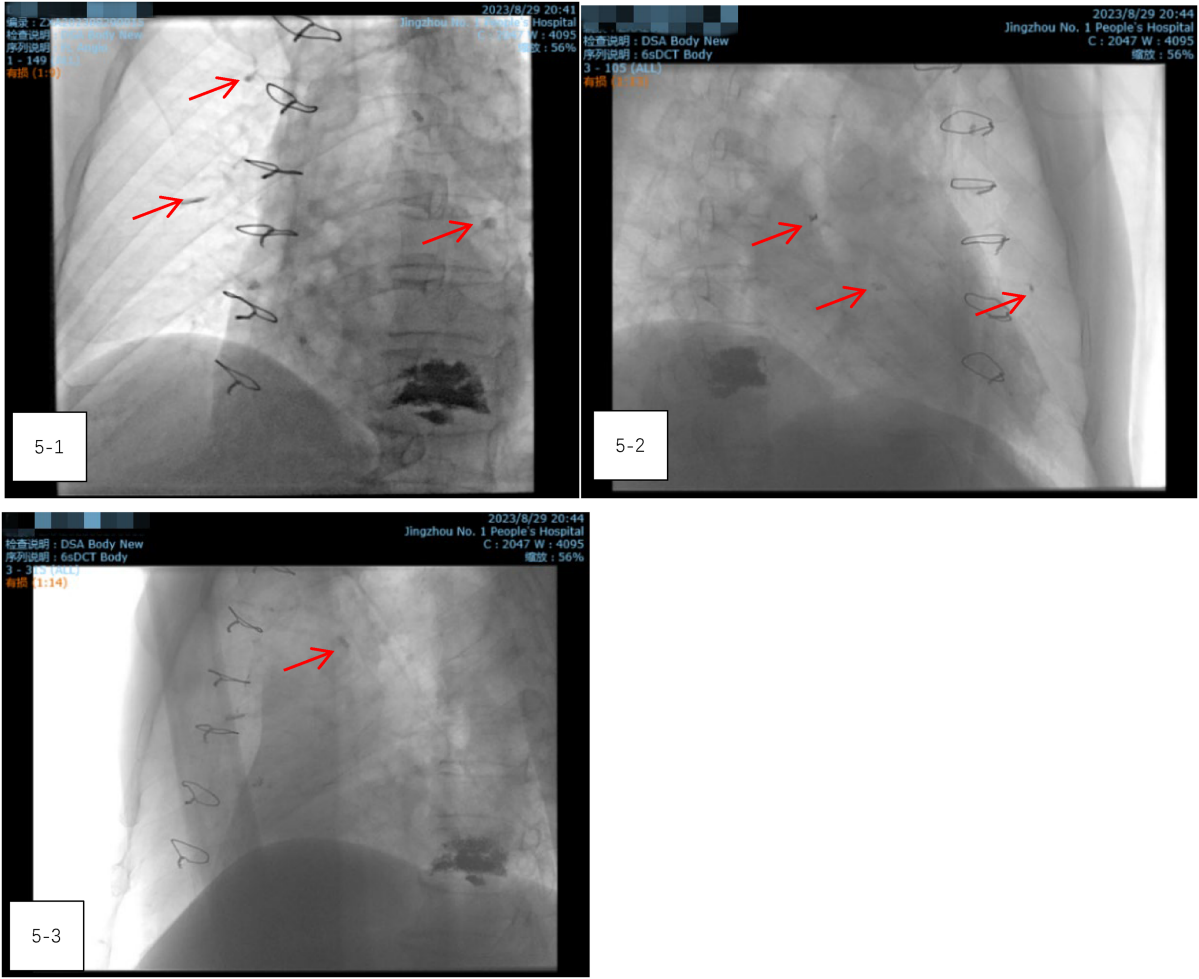
(5-1–5-3) Radiography showed a reduction in the size of the bone cement within the heart, but an increase in emboli within the pulmonary artery.

**Figure 6:**
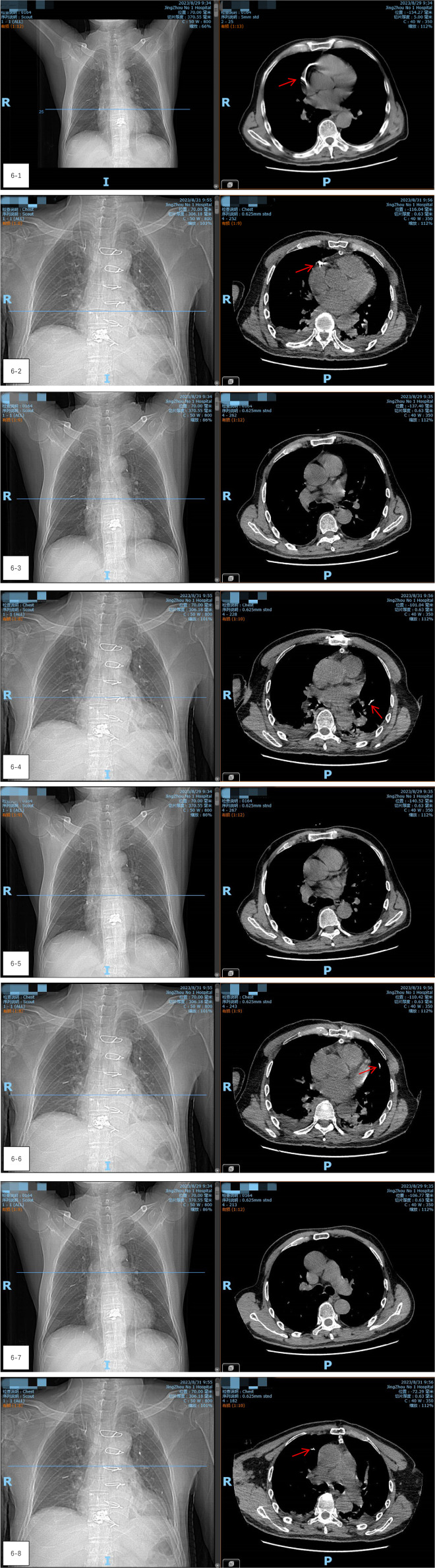
(6-1–6-8) A follow-up chest CT scan on the second day after thoracotomy, compared with the preoperative CT, confirmed a significant reduction in the high-density shadow in the heart and the appearance of new shadows in the lung, verifying the migration of fractured cement into the pulmonary artery.

**Figure 7:**
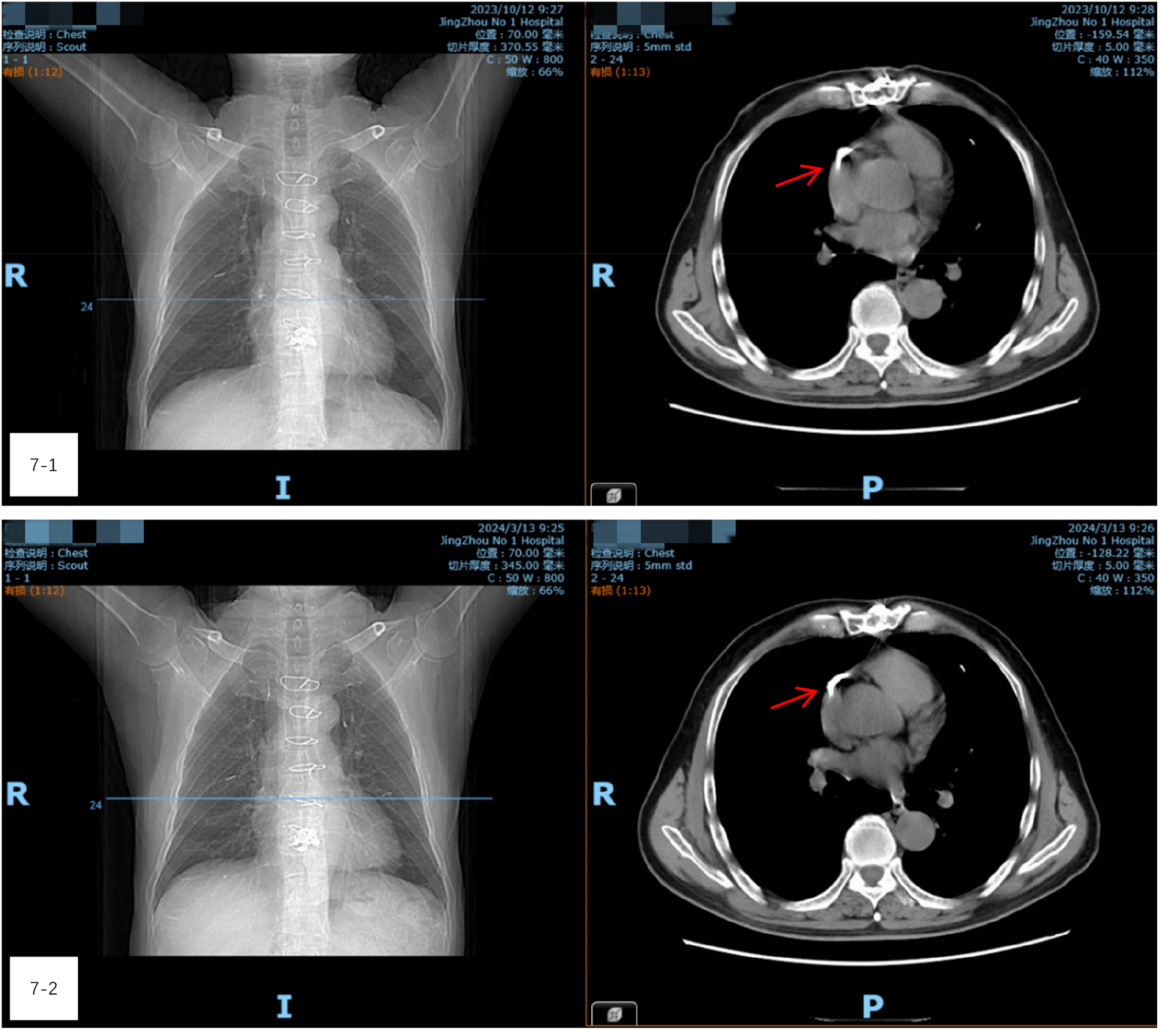
(7-1 and 7-2) Follow-up examinations one- and three-months post-surgery. CT scans showed no significant changes in the cement fragments within the heart and lungs, indicating stable conditions.

After closing the incision, the patient was transferred to the digital subtraction angiography (DSA) operating room. Radiography showed a reduction in the size of the bone cement within the heart, but an increase in emboli within the pulmonary artery (Figures 5-1–5-3). It was concluded that the bone cement had likely fractured during the operation and migrated into the pulmonary artery. A follow-up chest CT scan on the second day after thoracotomy, compared with the preoperative CT, confirmed a significant reduction in the high-density shadow in the heart and the appearance of new shadows in the lung, verifying the migration of fractured cement into the pulmonary artery (Figures 6-1–6-8).

On the third day following thoracotomy, the patient was able to walk without any noticeable delay in mobilization. His recovery proceeded smoothly, and he was successfully discharged. Follow-up examinations one- and three-months post-surgery (Figures 7-1, 7-2) revealed no symptoms such as chest tightness, shortness of breath, or discomfort near the heart. CT scans showed no significant changes in the cement fragments within the heart and lungs, indicating stable conditions.

## 3 Discussion

As the population ages, the incidence of osteoporotic vertebral compression fractures (OVCF) is on the rise, presenting a significant healthcare challenge (1). Minimally invasive surgical options, such as percutaneous kyphoplasty (PKP) and percutaneous vertebroplasty (PVP), have emerged as effective and safe interventions for stabilizing vertebral compression fractures. These procedures involve the injection of polymethyl methacrylate (PMMA), commonly referred to as bone cement, into the collapsed vertebra (6). By doing so, they not only alleviate pain but also reduce the likelihood of complications associated with immobility, including pressure sores, urinary infections, hypostatic pneumonia, and deep vein thrombosis in the lower limbs. Given their minimally invasive nature, which results in less trauma, reduced blood loss, and shorter operation times, PKP and PVP are often the preferred treatment for elderly patients who may have multiple underlying health issues. Despite their advantages, the potential complications of PKP and PVP cannot be overlooked. Local leakage of PMMA is a common occurrence, though it is usually asymptomatic (3).

S. Yom et al. categorized bone cement leakage into three types: type B (through basivertebral veins), type S (through segmental veins), and type C (through cortical defects). Notably, S-type and B-type leaks, which occur via segmental and paravertebral veins respectively, were found to be more prevalent than type C leaks (7). Such paravertebral vein leakage can lead to serious complications, including intracardiac bone cement embolism and pulmonary embolism (8). Once leakage occurs, the cement can migrate to the heart through the basivertebral vein and the extracorporeal vertebral venous plexus, and flows into the superior vena cava through the segmental vein, the great nerve root vein, the azygos vein, the semi-azygos vein and the accessory semi-azygos vein (9). In our case, a high-density, cable-like image was observed in the left anterior aspect of the T9 vertebra, suggesting that the flow of bone cement may have followed the segmental vein, semi-azygos vein, and superior azygos vein, ultimately reaching the heart and pulmonary artery.

The causes of bone cement leakage can generally be classified into three categories: (1) insufficient polymerization of the cement, (2) the placement of the puncture needle near or within the vertebral cortex or central venous groove, and (3) overfilling of the cement (8). Research indicates that high-viscosity cement (HVC) significantly reduces the incidence of leakage compared to low-viscosity cement (LVC), particularly in the disc space and venous systems, without compromising clinical outcomes (10). In our instance, the bone cement used was Eurofix884108; however, its viscosity was not specified in the instructions. The mixing time was noted to be 30 s at room temperatures of 20°C, 23°C, and 26°C, with an initial injection time of 2 min and 20 s, 2 min and 15 s, and 2 min, respectively.

While most patients with cement leakage remain asymptomatic, such leaks can increase the risk of cardiopulmonary embolism and nerve damage (11). In rare cases, migration of cement particles into the paravertebral venous system can result in embolization of the right ventricle and pulmonary artery (8).

If a bone cement embolus lodges in the right atrium or ventricle, it may cause cardiac perforation and pericardial tamponade. Studies have indicated that linear pieces of bone cement measuring 10–20 mm can lead to pericardial tamponade, while pieces exceeding 20 mm can cause right ventricular perforation (12). Thus, the possibility of cement embolization in the heart warrants serious attention. A retrospective study by Fadili Hassani et al. reported an incidence of intracardiac cement embolism following PVP of 3.9%, with symptomatic cases being even rarer, at approximately 0.3%. It was noted that multi-segmental cement injections during a single procedure were significant risk factors for these complications (13). Previous case reviews of intracardiac cement embolism highlighted symptoms such as chest pain, dyspnea, and shock, primarily resulting from the cement puncturing the heart and leading to pericardial tamponade (8). These symptoms must be distinguished from those of angina pectoris or acute myocardial infarction. When a patient presents with these symptoms following PKP or PVP, the possibility of a cardiac cement embolism should be considered.

Currently, there is no consensus on how to manage asymptomatic patients with intracardiac cement embolism; approaches can range from clinical observation to anticoagulation therapy (9, 14). For symptomatic patients, treatment options may include anticoagulation, pain management, and oxygen therapy. In severe cases, interventional procedures or open-heart surgery may be necessary to remove the embolus (15). In our case, the patient experienced transient chest discomfort, and due to the size of the bone cement exceeding 20 mm in the heart, which posed a risk of perforation, we opted for thoracotomy at the request of the patient and their family.

In conclusion, vascular leakage of bone cement presents a potentially fatal complication of PKP and PVP that warrants close attention. Prevention is crucial, and based on our case, we recommend the following strategies to minimize the risk of bone cement leakage: (1) Avoid excessive extension of the push rod, ensuring it does not exceed one-third of the anterior portion of the vertebral body; (2) Adhere strictly to the manufacturer's guidelines for bone cement preparation, including precise control of mixing ratios, and injection timings; and (3) Inject bone cement slowly to prevent excessive pressure that could lead to leakage. If cement leakage is suspected, a chest examination should be conducted to evaluate for potential intracardiac or pulmonary embolism, and a tailored treatment plan should be developed accordingly.

## Data Availability

The original contributions presented in the study are included in the article/Supplementary Material, further inquiries can be directed to the corresponding author.
